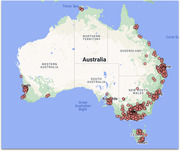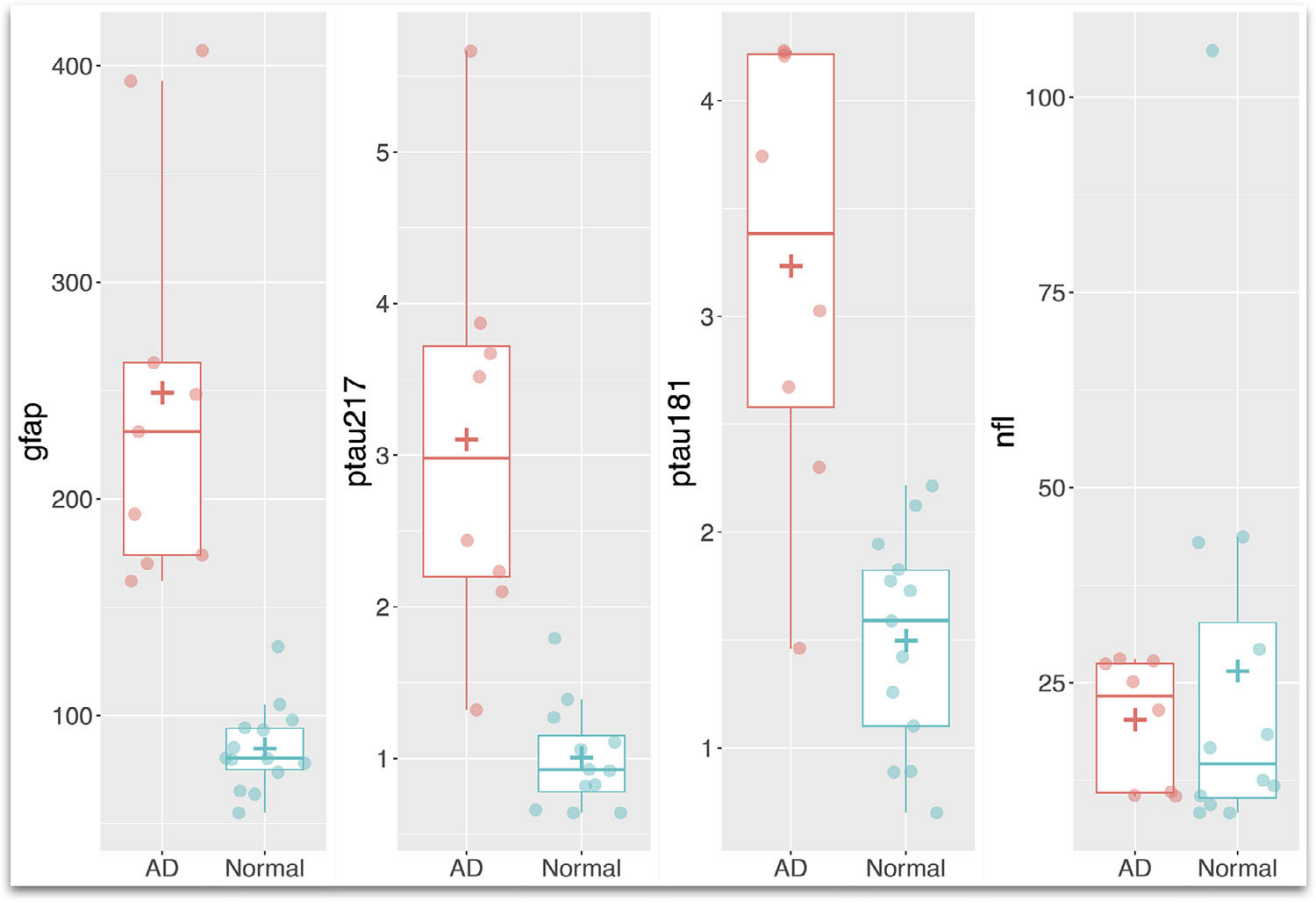# The Markers in Neuropsychiatric Disorders Study (The MiND Study): progress of a large nationwide Australian blood biomarker study and platform for collaborations to improve diagnosis and care of dementia and other brain conditions

**DOI:** 10.1002/alz.088415

**Published:** 2025-01-09

**Authors:** Dhamidhu Eratne, Courtney Lewis, Christa Dang, Jasleen Grewal, Matthew Kang, Henrik Zetterberg, Kaj Blennow, Alexander Santillo, Dennis Velakoulis, The MiND Study Group

**Affiliations:** ^1^ Neuropsychiatry, The Royal Melbourne Hospital, Parkville, VIC Australia; ^2^ University of Melbourne, Parkville, VIC Australia; ^3^ The University of Melbourne, Parkville, VIC Australia; ^4^ National Ageing Research Institute, Melbourne, VIC Australia; ^5^ Alfred Hospital, Melbourne, VIC Australia; ^6^ UCL Institute of Neurology, Queen Square, London UK; ^7^ Wisconsin Alzheimer's Disease Research Center, School of Medicine and Public Health, University of Wisconsin‐Madison, Madison, WI USA; ^8^ Hong Kong Center for Neurodegenerative Diseases, Clear Water Bay Hong Kong; ^9^ UK Dementia Research Institute at UCL, London UK; ^10^ Department of Psychiatry and Neurochemistry, Institute of Neuroscience and Physiology, the Sahlgrenska Academy at the University of Gothenburg, Mölndal Sweden; ^11^ Department of Psychiatry and Neurochemistry, Institute of Neuroscience and Physiology, The Sahlgrenska Academy at the University of Gothenburg, Mölndal Sweden; ^12^ Clinical Neurochemistry Laboratory Sahlgrenska University Hospital, Mölndal Sweden; ^13^ Clinical Memory Research Unit, Lund University, Malmö Sweden

## Abstract

**Background:**

The Markers in Neuropsychiatric Disorders Study (The MiND Study) is investigating the diagnostic and wider utility of blood based biomarkers such as neurofilament light chain (NfL), glial fibrillary acidic protein (GFAP), and phosphorylated tau (p‐tau), as well as other markers, to improve timely and accurate diagnosis of dementia and distinction from non‐neurodegenerative and primary psychiatric disorder (PPD). This in‐progress study has expanded significantly, becoming a robust platform for Australian and international collaborations.

**Methods:**

Participants have been recruited and blood samples collected across Australia. Longitudinal clinical/diagnostic, questionnaire, cognitive, and health utilisation data is collected. Second timepoint bloods (24 months) are now underway. Samples have been been analysed for NfL, GFAP, p‐tau217, p‐tau181, amyloid beta, and genetics.

**Results:**

As of January 2024, over 1500 referrals have been received, and over 1000 participants recruited from diverse specialist and community settings. Partnering with public and private pathology services, blood sample collection and storage processes have been developed across most of Australia. Approximately 2/3 of participants have volunteered to participate in the second timepoint blood samples. Findings thus far include plasma NfL reference ranges, strong diagnostic utility of blood and cerebrospinal fluid NfL to distinguish dementia from PPD, and GFAP and ptau217 to distinguish Alzheimer from non‐Alzheimer disease with very high accuracy, and high value placed on NfL testing by participants. Numerous collaborations and sub‐studies have developed, investigating cognitive and neuroimaging markers.

**Conclusions:**

The MiND Study is large biobank and platform supporting collaborations, pioneering research to lead to clinical translation, with particular focus on clinical translation in to broad clinical settings, younger onset dementia, primary care settings, and non‐AD and neurodegenerative mimics such as primary psychiatric disorders.